# Targeting Angiogenesis-Dependent Calcified Neoplasms Using Combined Polymer Therapeutics

**DOI:** 10.1371/journal.pone.0005233

**Published:** 2009-04-21

**Authors:** Ehud Segal, Huaizhong Pan, Paula Ofek, Taturo Udagawa, Pavla Kopečková, Jindřich Kopeček, Ronit Satchi-Fainaro

**Affiliations:** 1 Department of Physiology and Pharmacology, Sackler School of Medicine, Tel Aviv University, Tel Aviv, Israel; 2 Department of Pharmaceutics and Pharmaceutical Chemistry, Center for Controlled Chemical Delivery, University of Utah, Salt Lake City, Utah, United States of America; 3 Vascular Biology Program and Department of Surgery, Karp Family Research Laboratories, Children's Hospital Boston and Harvard Medical School, Boston, Massachusetts, United States of America; Cleveland Clinic, United States of America

## Abstract

**Background:**

There is an immense clinical need for novel therapeutics for the treatment of angiogenesis-dependent calcified neoplasms such as osteosarcomas and bone metastases. We developed a new therapeutic strategy to target bone metastases and calcified neoplasms using combined polymer-bound angiogenesis inhibitors. Using an advanced “living polymerization” technique, the reversible addition-fragmentation chain transfer (RAFT), we conjugated the aminobisphosphonate alendronate (ALN), and the potent anti-angiogenic agent TNP-470 with N-(2-hydroxypropyl)methacrylamide (HPMA) copolymer through a Glycine-Glycine-Proline-Norleucine linker, cleaved by cathepsin K, a cysteine protease overexpressed at resorption sites in bone tissues. In this approach, dual targeting is achieved. Passive accumulation is possible due to the increase in molecular weight following polymer conjugation of the drugs, thus extravasating from the tumor leaky vessels and not from normal healthy vessels. Active targeting to the calcified tissues is achieved by ALN's affinity to bone mineral.

**Methods and Finding:**

The anti-angiogenic and antitumor potency of HPMA copolymer-ALN-TNP-470 conjugate was evaluated both *in vitro* and *in vivo*. We show that free and conjugated ALN-TNP-470 have synergistic anti-angiogenic and antitumor activity by inhibiting proliferation, migration and capillary-like tube formation of endothelial and human osteosarcoma cells *in vitro*. Evaluation of anti-angiogenic, antitumor activity and body distribution of HPMA copolymer-ALN-TNP-470 conjugate was performed on severe combined immunodeficiency (SCID) male mice inoculated with mCherry-labeled MG-63-Ras human osteosarcoma and by modified Miles permeability assay. Our targeted bi-specific conjugate reduced VEGF-induced vascular hyperpermeability by 92% and remarkably inhibited osteosarcoma growth in mice by 96%.

**Conclusions:**

This is the first report to describe a new concept of a narrowly-dispersed combined polymer therapeutic designed to target both tumor and endothelial compartments of bone metastases and calcified neoplasms at a single administration. This new approach of co-delivery of two synergistic drugs may have clinical utility as a potential therapy for angiogenesis-dependent cancers such as osteosarcoma and bone metastases.

## Introduction

There is an unmet medical need for new targeted treatments for bone neoplasms such as bone metastases and osteosarcoma. Approximately 60%–84% of patients diagnosed with solid tumors develop bone metastases. Prostate, breast, lung, kidney, and thyroid cancers account for 80% of skeletal metastases [1]. Out of the bone neoplasms, osteosarcoma, is the most common type of primary bone cancer and classified as a malignant mesenchymal neoplasm in which the tumor directly produces impaired osteoid [2]. It is a highly vascular and extremely destructive malignancy that most commonly arises in the metaphyseal ends of long bones [3]. Over the past two decades, multimodality treatment consisting of aggressive chemotherapy combined with radical surgical resection, has been the mainstay of osteosarcoma management, with achievable 5 year survival rates of 50 to 70% in patients who do not have metastatic disease at presentation. Several strategies were proposed [4], however, still one-third of the patients die from this devastating cancer, and for those with unresectable disease there are no curative systemic therapies.

Tumor progression and metastases are highly dependent on oxygen and nutrients supplied by new angiogenic blood vessels which formation is stimulated by the tumor itself and its environment [5]. Anti-angiogenic therapy combined with conventional treatment holds great potential for osteosarcoma management and metastatic risk reduction. There is a vast amount of preclinical and clinical data suggesting that anti-angiogenics are effective treatments for osteosarcomas [4], [6], [7] and for bone metastases originating from prostate and breast cancers [8], [9], [10]. Several angiogenesis inhibitors have been recently approved for clinical use [11], [12]. Angiogenesis inhibitors, such as TNP-470 [13] and its non-toxic N-(2-hydroxypropyl)methacrylamide (HPMA) copolymer-conjugated form, caplostatin [13], [14], are emerging as a new modality of anticancer drugs. Nonetheless, synthesizing new targeted angiogenesis inhibitors, optimizing their dose and determining the best combinations of novel angiogenesis inhibitors remain formidable challenges. These hurdles may limit the use of a single anti-angiogenic agent. Another noteworthy obstacle is that the vast majority of clinically used anticancer and anti-angiogenic drugs are small molecules that exhibit a short half-life in the bloodstream and a high overall clearance rate. These low-molecular weight drugs diffuse rapidly into healthy tissues and are distributed evenly within the body. As a consequence, relatively small amounts of the drug reach the target site, and therapy is associated with low efficacy and severe side effects [15], [16].

Here we describe the synthesis and characterization of a novel conjugate of HPMA copolymer, TNP-470 and the aminobisphosphonate, alendronate (ALN). TNP-470 is a low molecular weight synthetic analogue of fumagillin able to selectively inhibit angiogenesis and suppress tumor growth [17]. In clinical trials, it slowed tumor growth in patients with metastatic cancer. However, at higher doses necessary for tumor regression, many patients experienced toxicity [18]. Because of dose limiting neurotoxicity, TNP-470 has been stalled in Phase II. Due to its strong potency, poor pharmacokinetic profile, and poor solubility, TNP-470 is an ideal candidate for polymer conjugation. Thus, to apply this drug clinically to more effectively treat tumors, drug targeting to tumor tissue is necessary to increase site specificity and reduce side effects. We have recently shown that caplostatin, an HPMA copolymer conjugate of TNP-470 prolonged the circulating life of the drug, increased the accumulation of the drug in angiogenic tissue through the enhanced permeability and retention (EPR) effect [19] and prevented it from crossing the blood brain barrier (BBB), therefore abrogating neurotoxicity [13], [20], [21]. Several other TNP-470 analogs [22], [23], [24] or alternatively Methionine-aminopeptidase-2 (MetAP2) inhibitors [25], [26], [27], [28] have been recently developed with the aim of “resurrecting” TNP-470 [29].

Bisphosphonates (BPs) are analogues of inorganic pyrophosphate, an endogenous regulator of bone mineralization [30], [31]. Several BPs are effective treatments in osteoporosis, Paget's disease of bone, myeloma, and bone metastases [32]. BPs have been shown to inhibit angiogenesis [33], [34]. Furthermore, there is a vast amount of literature on the use of BPs for the treatment of bone metastases originating from prostate and breast cancers [9], [35], [36], [37], [38]. Their strong affinity to bone mineral [39], their low toxicity and anti-angiogenic activity make the BPs excellent candidates for targeting calcified neoplasms. There are several studies and publications describing the conjugation of BPs directly to cancer treating agents like antineoplastics, radionucleotides or nucleoside analogs for the treatment of bone metastases [40]. ALN is a nitrogen-containing BP drug approved by the FDA that acts as a specific inhibitor of osteoclast-mediated bone resorption and several other bone diseases [41], [42]. ALN is considered one of the potent BP for the treatment of bone related diseases and cancer-associated hypercalcemia. It was shown to have antitumor effect in several in vivo cancer models through several different mechanisms [43], [44], [45]. In addition, ALN was found to have anti-angiogenic activity through (i) suppression of VEGF-induced Rho activation in an ovarian cancer model [46], (ii) inhibition of a key enzyme, farnesyl pyrophosphate synthase, in the mevalonate pathway, thereby preventing the biosynthesis of isoprenoid compounds that are essential for the posttranslational modification of small guanosine triphosphate (GTP)-binding proteins such as Rab, Rho, and Rac [47]; and (iii) regulation of cellular level of MMP-2 expression in osteosarcoma cell lines [48]. ALN bears a primary amine which facilitates conjugation with HPMA copolymer through a cathepsin K-cleavable linker, glycine-glycine-proline-norleucine (Gly-Gly-Pro-Nle). Cathepsin K is involved in bone resorption and its expression is stimulated by inflammatory cytokines that are released after tissue injury and in bone neoplasms [12], [49]. We propose that combined therapy of an HPMA copolymer-ALN-TNP-470 conjugate, can be used as a preventive or curative new concept of anti-angiogenic and antitumor treatment. A major benefit of polymer-targeted angiogenesis inhibitors is reduced toxicity compared to conventional cytotoxic agents [50]. Consequently, beside their use in clinically evident disease, our targeted polymer therapeutics may potentially be used for the treatment of asymptomatic individuals who are at risk for relapse of ostesarcoma, breast and prostate cancer metastases. This approach may lead to a major paradigm shift in cancer treatment from current methods where treatment is generally not initiated until the cancer becomes clinically evident [13].

## Materials and Methods

### Materials

Cathepsin K inhibitor III was purchased from Calbiochem, Germany. Phalloidin-TRITC conjugate, Propidium iodide (PI) and hydroxyapatite (HA) were purchased from Sigma-Aldrich, Israel. ALN was from Alcon Biosciences, India. Ultra pure double distilled water (DDW) was purchased from Biological Industries, Israel. Antifade® mounting medium was from Biomeda Corp. Alexa® Fluor 594 human transferrin was from Molecular Probes™. Peroxidase Block was purchase from Merck, Germany. Primary rat anti-murine CD34 antibody (MEC 14.7) was from Abcam, (Cambridge, MA). Rabbit anti-rat antibody, anti-rabbit horseradish peroxidase-conjugated antibody (ABC detection kit) and ImmPACT™ DAB diluent kit were from Vector Laboratories, CA, USA. Boyden chambers 8 µm were from Transwell-Costar Corp. Hema 3 Stain System was from Fisher Diagnostics. EGM-2 medium was from Cambrex, USA and endothelial cells growth supplement (ECGS) from Zotal, Israel. All other chemical reagents, including salts and solvents, were purchased from Sigma-Aldrich. All reactions requiring anhydrous conditions were performed under an Ar or N_2_ atmosphere. Chemicals and solvents were either A.R. grade or purified by standard techniques.

### Cell culture

Saos-2 and MG-63-Ras human osteosarcoma cells were obtained from the American Type Culture Collection (ATCC). MG-63-Ras cells were transfected with activated *ras* (MG-63-Ras) as previously described in order to generate an *in vivo* fast-growing tumor cell line [51]. Cells were cultured in Dulbecco's modified Eagle's medium (DMEM) supplemented with 10% fetal bovine serum (FBS), 100 µg/ml Penicillin, 100 U/ml Streptomycin, 12.5 U/ml Nystatin and 2 mM L-glutamin (Biological Industries, Israel). Cells were grown at 37°C in 5% CO_2_. mCherry-labeled MG-63-Ras human osteosarcoma cell line was obtained by infection with a pQC-mCherry retroviral vector. The infected cells were selected for stable expression of mCherry using puromycin. Human umbilical vein endothelial cells (HUVEC) were purchased from Lonza, Switzerland. HUVEC were cultured in EGM-2 medium (Lonza, Switzerland) and were grown at 37°C; 5% CO_2_.

### Methods

#### Ethics Statement

All animal procedures were performed in compliance with Tel Aviv University, Sackler School of Medicine guidelines and protocols approved by the Institutional Animal Care and Use Committee. Mice's body weight and tumor size were measured three times a week.

### Generation of mCherry-infected MG-63-Ras human osteosarcoma cell line

mCherry was subcloned from pART7-mCherry (kindly provided by A. Avni from Tel Aviv University), into pQCXIP (Clontech). Human embryonic kidney 293T (HEK 293T) cells were co-transfected with pQC-mCherry and the compatible packaging plasmids (pMD.G.VSVG and pGag-pol.gpt). Forty eight hours following transfection, the pQC-mCherry retroviral particles containing supernatant was collected. MG-63-Ras human osteosarcoma cells were infected with the retroviral particles media, and 48 h following the infection, mCherry positive cells were selected by puromycin resistance.

### Cell proliferation assay

HUVEC were plated at 10,000 cells/well onto 24-well culture plates in EBM-2 supplemented with 5% FBS and incubated for 24 h (37°C; 5% CO_2_). Medium was replaced with 2.5% EBM-2 supplemented with 1% ECGS. Human osteosarcoma Saos-2 or MG-63-Ras cells were plated at 2500 cells/well in DMEM supplemented with 5% FBS and incubated for 24 h (37°C; 5% CO_2_). The medium was then replaced with DMEM supplemented with 10% FBS. Cells were exposed to ALN, TNP-470, and HPMA copolymer-ALN-TNP-470 conjugate or with equivalent concentrations of combinations of free ALN and TNP-470 at serial dilutions. HUVEC were incubated with or without 1 µM of cathepsin-K inhibitor III. Control cells were grown in the presence or absence of growth factors. HUVEC or Saos-2 viable cells were counted by a Z1 Coulter® Particle Counter (Beckman Coulter™) or by XTT reagent respectively after 72 h of incubation.

### Isobolograms of ALN-TNP-470 drug combination treatments

IC_50_ represents the concentration of a drug that is required for 50% inhibition *in vitro*. The IC_30, 50, 70_ values of treatment with ALN, TNP-470 and their respective combinations were calculated from HUVEC proliferation assay. IC_30, 50, 70_ values of TNP-470 and ALN were marked on X, Y axis respectively and a line which represents additive effect was drawn between each inhibitory concentration (IC). The combination index of each treatment was calculated according to the classic isobologram equation combination index = [(D)_1_/(Dx)_1_]+[(D)_2_/(Dx)_2_] as previously described [52]. Area on the right side of each IC additive line represents antagonist effect while the left side represents synergic effect.

### Synthesis of HPMA copolymer-ALN-TNP-470 conjugate

#### Synthesis of ALN containing monomer (MA-Gly-Gly-Pro-Nle-ALN)

The monomer was synthesized as described previously [27]. Briefly, MA-Gly-Gly-Pro-Nle was synthesized by solid phase peptide synthesis (SPPS) and manual Fmoc/tBu strategy using 2 g of 2-chlorotrityl chloride beads with 80% of loading leading to a yield of 0.88 g, 95%. MA-Gly-Gly-Pro-Nle-OH (100 mg, 0.24 mmol) and 2-mercaptothiazoline (TT, 33 mg, 0.28 mmol) were dissolved in a mixture of 2 ml 1,4-dioxane and 1 ml tetrahydrofuran (THF), and cooled to 4°C. DCC (60 mg, 0.29 mmol) in 1 ml of 1,4-dioxane was added dropwise and the reaction mixture stirred overnight at 4°C. After the reaction, DCU was removed by filtration and the filtrate was added to ALN aqueous solution (70 mg, 4 ml; pH was adjusted to ∼7.4 by NaHCO_3_ solution). The reaction mixture was stirred overnight at room temperature. The solvent was removed on a rotary evaporator, the residue was re-dissolved in water and extracted with ethyl acetate 3 times to remove TT. The product was purified by preparative high performance liquid chromatography (HPLC) with a yield of 83 mg. The MALDI-TOF spectrum negative ion: m/z = 640 (M−H^+^), 662 (M-mono-Na salt−H^+^); positive ion: m/z = 642 (M+H^+^), 664 (M-mono-Na salt+H^+^), 686 (M-mono-Na salt+Na^+^).

#### Synthesis of amine containing monomer (MA-Gly-Gly-Pro-Nle-NH(CH_2_)_2_NH_2_)

MA-Gly-Gly-Pro-Nle-NH(CH_2_)_2_NH_2_ was synthesized by SPPS using 1.5 g of 2-chlorotrityl chloride beads. First, 6 times excess of ethylenediamine in anhydrous THF was applied, followed by Fmoc-amino acids, and capping with MA-Gly-Gly-OH. The final peptide was cleaved by 5% TFA in dichloromethane (DCM) with a yield of 0.85 g, 89%. The purity of product was proved by HPLC (buffer A: H_2_O 0.1% TFA, buffer B: acetonitrile 0.1%TFA; gradient method: buffer B 2%–60%/30 min; 1 ml/min; single peak, elution time 8.27 min). The MALDI-TOF spectrum (Fmoc derivative) positive ion: m/z = 697 (M+Na^+^), 713 (M+K^+^).

#### Synthesis FITC containing monomer (MA-FITC)

Fluoresceinisothiocyanate (FITC) (1 g, 2.57 mmol) and *N*-(3-aminopropyl) methacrylamide hydrochloride (0.92 g, 5.14 mmol) were dissolved in 5 ml dimethylformamide (DMF) and cooled to 4°C, then *N,N*-diisopropylethylamine (DIPEA) (1.79 ml, 10.3 mmol) in 2 ml of DMF was added dropwise. The reaction mixture was stirred at 4°C for 2 days. Then, the reaction mixture was poured into 100 ml water (pH∼4–5) and pH adjusted to ∼4 by 6 N HCl. The precipitate was filtered off, washed with water, and vacuum dried over P_2_O_5_.

#### Synthesis of polymer precursor containing ALN, NH_2_, and (optionally) FITC groups (HPMA copolymer-ALN-NH_2_)

MA-Gly-Gly-Pro-Nle-ALN (73 mg), MA-Gly-Gly-Pro-Nle-NH(CH_2_)_2_NH_2_ (55 mg), HPMA (200 mg), MA-FITC (0 mg or 4 mg) and 4,4′-azobis(4-cyanovaleric acid) (V-501, 3 mg) as the initiator were dissolved in 2 ml of water. The solution was bubbled with N_2(g)_ for 10 min, the ampoule sealed, and the mixture polymerized at 60°C for 24 h.

Alternatively, we used reversible addition-fragmentation chain transfer (RAFT) polymerization technique. MA-Gly-Gly-Pro-Nle-ALN (293 mg), MA-Gly-Gly-Pro-Nle-NH(CH_2_)_2_NH_2_ (215 mg), HPMA (948 mg), MA-FITC (0 mg or 4 mg), 2,2′-Azobis[2-(2-imidazolin-2-yl)propane]dihydrochloride (VA-044 1.6 mg) as initiator and *S*,*S*′-bis(α,α′-dimethyl-α″-acetic acid) trithiocarbonate as chain transfer agent (TTC 4.2 mg) were dissolved in 7.5 ml of water. The solution was bubbled with N_2(g)_ for 30 min, sealed in ampoule , and the mixture polymerized at 30°C for 48 h. Both resulting polymers were purified by dissolving in water and precipitating into an excess of acetone; following each precipitation, the precipitate was washed with acetone. Finally, the polymers were dissolved in 15 ml of water; pH adjusted to 12 with 1 N NaOH, and dialyzed against DI water for 24 h at 4°C (MWCO 12-14 kDa) to remove ALN monomer. The samples were freeze-dried after dialysis.

#### Binding of TNP-470 to HPMA copolymer-ALN

HPMA copolymer-ALN-NH_2_ (150 mg) was dissolved in 6 ml of DMF (if necessary, a small amount of water was added to dissolve the polymer) and cooled to 4°C. Then, TNP-470 (150 mg) in 1 ml DMF was added. The reaction mixture was stirred at 4°C in dark for 12 h. Following the reaction, the conjugate was precipitated into acetone and purified by reprecipitation (3 times) from an aqueous solution into an excess of acetone. The precipitate was washed with acetone and the residue was dissolved in water and dialyzed for 1 day at 4°C (MWCO 12–14 kDa) against DI water. The conjugate was isolated by freeze-drying.

### Characterization of HPMA copolymer-ALN-TNP-470 conjugate (Scheme S1)

#### Determination of ALN content

The formation of chromophoric complex between ALN and Fe^3+^ ions in perchloric acid solution was used to determine the ALN content by spectrophotometry [53]. Briefly, 0.1 ml conjugate (conc. 2–10 mg/ml) was mixed with 0.1 ml 4 mM FeCl_3_ and 0.8 ml 0.2 M HClO_4_ and absorbance at 300 nm was measured against blank. The calibration curve was prepared by using ALN solution at concentration range 0–3 mM.

### Estimation of TNP-470 content

The content of TNP-470 was estimated from the content of NH_2_ groups in the HPMA copolymer-ALN-NH_2_ precursor (Scheme S1). It was assumed that the TNP-470 binding was quantitatively equal. The content of NH_2_ groups was determined by ninhydrin method using an amine containing monomer (N-(3-aminopropyl) methacrylamide) as the calibration sample [modified from [45]].

### Determination of FITC content

The content of FITC was measured spectrophotometrically using ε 80000 M- 1 cm-1 in 0.1 M borate buffer (Scheme S1).

### Determination of HPMA copolymer-ALN-TNP-470 conjugate molecular weight and polydispersity index (PDI)

Determination of polymer-drug molecular weight profile by size exclusion chromatography (SEC) was done using AKTA/FPLC system (Pharmacia/GE Healthcare), Superose 12 HR10/30 column in 0.1 M acetate buffer pH 5.5+30% acetonitrile, pH 5.5, flow rate 0.4 ml/min; UV and RI detection. Weight average molar mass (M_w_) was evaluated from the SEC profiles and the PDI was calculated according to the formula M_w_/M_n_. The column was calibrated using polyHPMA fractions of narrow polydispersity.

### Quantitative evaluation of HPMA copolymer-ALN-TNP-470 conjugate size distribution

The mean hydrodynamic diameter of the conjugate was evaluated using a real time particle analyzer (NanoSight LM20™) containing a solid-state, single mode laser diode (<20 mW, 655 nm) configured to launch a finely focused beam through a 500 µl sample chamber. HPMA copolymer-ALN-TNP-470 conjugate was dissolved in phosphate buffered saline (PBS) to final concentrations of 0.5, 1 and 2 mg/ml. The samples were then injected into the chamber by syringe and allowed to equilibrate to unit temperature (23°C) for 30 sec. The particles dynamics were visualized at 30 frames per second (fps) for 60 sec at 640×480 resolution by the coupled charge device (CCD) camera. The paths the particles take under Brownian motion over time were analyzed using Nanoparticle Tracking Analysis (NTA®) software. The diffusion coefficient and hence sphere equivalent hydrodynamic diameter of each particle was separately determined and the particle size distribution profiles were generated. Each sample was measured three times in triplicates, and the results represent the mean diameter.

### Hydroxyapatite (HA) binding assay

In order to assess the ability of HPMA copolymer-ALN-TNP-470 conjugate to bind to bone mineral its binding potency to HA was evaluated. HPMA copolymer-ALN-TNP-470 conjugate was dissolved in PBS, pH 7.4 (1 mg/ml). The conjugate solution (500 µl) was incubated with HA powder (15 mg), in 500 µl PBS, pH 7.4. HPMA copolymer-Gly-Phe-Leu-Gly was used as control. Incubated samples were centrifuged at 6000 RPM for 3 min and a sample from the upper layer (100 µl) was collected at selected time points. FPLC analysis using HiTrap desalting column (Amersham®) was used for detection of unbound conjugate in the samples (FPLC conditions: AKTA™ Purifier®, mobile phase 100% DDW, 2 ml/min, 215 nm). HA-binding kinetic analysis of the conjugate was performed using the Unicorn® AKTA™ software. Areas under the curve (AUC) were calculated from chromatographs at each time point. AUC of each HA-incubated conjugate chromatogram was normalized to percent AUC of conjugate sample in the absence of HA used as control.

### Intracellular trafficking of HPMA copolymer-ALN-TNP-470 conjugate

For all experiments, HUVEC and Saos-2 human osteosarcoma cells were seeded on sterile 13 mm cover glasses in 35 mm culture dishes 24 h before incubation with FITC-labeled HPMA copolymer-ALN-TNP-470 conjugate. HUVEC and Saos-2 cells were incubated with 10 µM HPMA copolymer-ALN-TNP-470 conjugate for 12 h, then washed several times with cold PBS, fixed with 3.5% paraformaldehyde for 15 min at room temperature (RT) and washed with PBS again. For counter staining, cells were permeabilized with 0.1% Triton-X100 for 3 min and rinsed with PBS again. For confocal imaging of FITC-labeled HPMA copolymer-ALN-TNP-470 conjugate cellular uptake by HUVEC, nuclei were labeled using propidium iodide (10 µg/ml) and cover glasses were mounted by Antifade® mounting media. Alternatively, actin filaments were labeled using phalloidin-TRITC conjugate (50 µg/ml, 40 min at RT) and cover glasses were mounted by Vectashild® DAPI containing medium. For conjugate endosomal pathway internalization analysis, HUVEC and Saos-2 cells were incubated with 10 µM HPMA copolymer-ALN-TNP-470 conjugate for 6 h. Following incubation, cells were washed several times with cold PBS, starved for 45 min in serum-free medium at 37°C and incubated with 40 µg/ml Alexa® Fluor 594 human transferrin for 1 h at 37°C. Cells were then fixed and mounted as described before. All slides were kept at 4°C in dark until confocal microscopy analysis was preformed.

### Confocal microscopy

Cellular uptake, internalization and colocalization of FITC-labeled HPMA copolymer-ALN-TNP-470-FITC conjugate were monitored utilizing a Zeiss Meta LSM 510 and a Leica TCS SP5 confocal imaging systems with 60× oil objectives. All images were taken using a multi-track channel acquisition to prevent emission cross-talk between fluorescent dyes. Single XY, XZ plane-images were acquired in 1024×1024 resolution. Images from Z stack acquisition were processed as separate channels using Huygens® deconvolution software and merged as a single image.

### HUVEC migration assay

Cell migration assays were performed using modified 8 µm Boyden chambers coated with 10 µg/ml fibronectin. HUVEC (15×10^4^ cells/100 µl) were exposed to HPMA copolymer-ALN-TNP-470 conjugate or to combinations of free ALN and TNP-470 at equivalent concentration and were added to the upper chamber of the transwell. Following 4 h of incubation, cells were allowed to migrate to the underside of the chamber for another 4 h in the presence or absence of vascular endothelial growth factor (VEGF) (20 ng/ml) in the lower chamber. Cells were then fixed with ice-cold methanol and stained (Hema 3 Stain System). The stained migrated cells were imaged using Nikon TE2000E inverted microscope integrated with Nikon DS5 cooled CCD camera by 10× objective, brightfield illumination. Migrated cells from the captured images per membrane were counted using NIH image software. Migration was normalized to percent migration, with 100% representing migration to VEGF alone.

### Capillary-like tube formation assay

The surface of 24-well plates was coated with Matrigel® basement membrane (50 µl/well; 10 mg/ml) on ice and was allowed to polymerize at 37°C for 30 min. HUVEC (3×10^4^ cells) were exposed to HPMA copolymer-ALN-TNP-470 conjugate or with combinations of free ALN and TNP-470 at equivalent concentrations and were seeded on coated plates in the presence of complete EGM-2 medium. Following 8 h of incubation (37°C; 5% CO_2_), cells were imaged using Nikon TE2000E inverted microscope integrated with Nikon DS5 cooled CCD camera by 4× objective, brightfield illumination. Images were analyzed for total tube area using Nikon NIS® elements image software.

### Miles vascular permeability assay

Balb/c male mice were injected subcutaneously (s.c.) with TNP-470, HPMA copolymer-ALN-TNP-470 conjugate (30 mg/kg TNP-470 equivalents) or saline (n = 5 mice/group). Three days later, a modified Miles assay was performed as previously described [54], [55]. Briefly, Evans blue dye (100 µl of a 1% solution in 0.9% NaCl) was injected into the retro-orbital plexus of the mice. Ten minutes later, 50 µl of human VEGF**_165_** (1 ng/µl) or PBS were injected intradermally into the pre-shaved back skin. Twenty minutes later, the animals were killed, and an area of skin that included the entire injection site was removed. Evans blue dye was extracted from the skin by incubation with formamide for 5 days at room temperature, and the absorbance of the extracted dye was measured at 620 nm. Data is expressed as mean±standard error of the mean (s.e.m.).

### Evaluation of antitumor activity of HPMA copolymer-ALN-TNP-470 conjugate

SCID male were inoculated s.c. with 5×10^5^ mCherry-labeled MG-63-Ras human osteosarcoma. Mice bearing 70 mm^3^ tumors were injected s.c. with combination of free ALN and TNP-470 (1∶1, 30 mg/kg), FITC-labeled HPMA copolymer-ALN-TNP-470 conjugate (30 mg/kg q.o.d.×3 times TNP-470-equivalent dose) or saline (n = 5 mice/group). Therapy was initiated at a relatively early state (70 mm^3^) in order to imitate a metastatic scenario as well as an early primary osteosarcoma. Tumor progression was monitored by caliper measurement (width×length^2^×0.52) and by CRI™ Maestro non-invasive intravital imaging system. At termination, tumors were dissected, weighed and analyzed. Data is expressed as mean±standard error of the mean (s.e.m.).

### Intravital non-invasive imaging of mCherry-labeled MG-63-Ras tumor-bearing mice and FITC-labeled HPMA copolymer-ALN-TNP-470 conjugate biodistribution in mice

CRI Maestro™ non-invasive fluorescence imaging system was used to follow tumor progression of mice bearing mCherry-labeled MG-63-Ras human osteosarcoma tumors and for biodistribution studies of FITC-labeled HPMA copolymer-ALN-TNP-470 conjugate. Mice were maintained on a non-fluorescent diet (Harlan) for the whole period of the experiment. Mice were anesthetized using ketamine (100 mg/kg) and xylazine (12 mg/kg), treated with a depilatory cream (Veet®) and placed inside the imaging system. Alternatively, selected organs from mice were dissected and placed inside the imaging system. Multispectral image-cubes were acquired through 550–800 nm spectral range in 10 nm steps using excitation (575–605 nm) and emission (645 nm longpass) filter set. Mice autofluorescence and undesired background signals were eliminated by spectral analysis and linear unmixing algorithm. Additionally, dissected tumors were fixed in 4% PFA and imaged as whole-mount by confocal microscopy as described earlier in order to assess the conjugate accumulation in the tumor site.

### Immunohistochemistry

Immunohistochemistry of tumor nodules was performed using 5 µm thick formalin-fixed, paraffin-embedded tissue sections. Paraffin sections were de-paraffinized, rehydrated, and stained by hematoxylin and eosin (H & E). For CD34 staining, slides were deparaffinized and pre-treated with 10 mM citrate, pH 6.0 for 50 min in a steam pressure cooker (Decloaking Chamber, BioCare Medical, Walnut Creek, CA). All further steps were performed at RT in a hydrated chamber. Slides were covered with Peroxidase Block (Merck, Germany) for 5 min to quench endogenous peroxidase activity, followed by incubation with 10% of rabbit serum in 50 mM Tris-HCl, pH 7.4, for 30 min to block non-specific binding sites. Primary rat anti-murine CD34 antibody (MEC 14.7 1∶50 dilution; Abcam, Cambridge, MA) was applied in 1% rabbit serum in Tris-HCl, pH 7.4 at RT for 1 h. Slides were washed in 50 mM TrisHCl, pH 7.4 and rabbit anti-rat antibody (1∶750 dilution; Vector Laboratories, CA, USA) was applied for 30 min, followed by anti-rabbit horseradish peroxidase-conjugated antibody (ABC detection kit, Vector Laboratories, CA, USA). Following further washing, immunoperoxidase staining was developed using a ImmPACT™ DAB diluent kit (Vector Laboratories, CA, USA) per the manufacturer instructions and counterstained with methyl green. Microvessel density (MVD) was calculated as previously described [56].

### Statistical methods

*In vitro* data from proliferation assays on HUVEC, MG-63-Ras and Saso-2 cells, HUVEC's migration and capillary-like tube formation expressed as mean±standard deviation (s.d.). *In vivo* data of Miles assay and evaluation of antitumor activity of HPMA copolymer-ALN-TNP-470 conjugate are expressed as mean±standard error of the mean (s.e.m.). Statistical significance was determined using an unpaired *t*-test. *P*<0.05 was considered statistically significant. All statistical tests were two-sided.

## Results

### Combination treatment of ALN and TNP-470 has synergistic inhibitory effect on proliferation of endothelial cells *in vitro*

**Figure 1:**
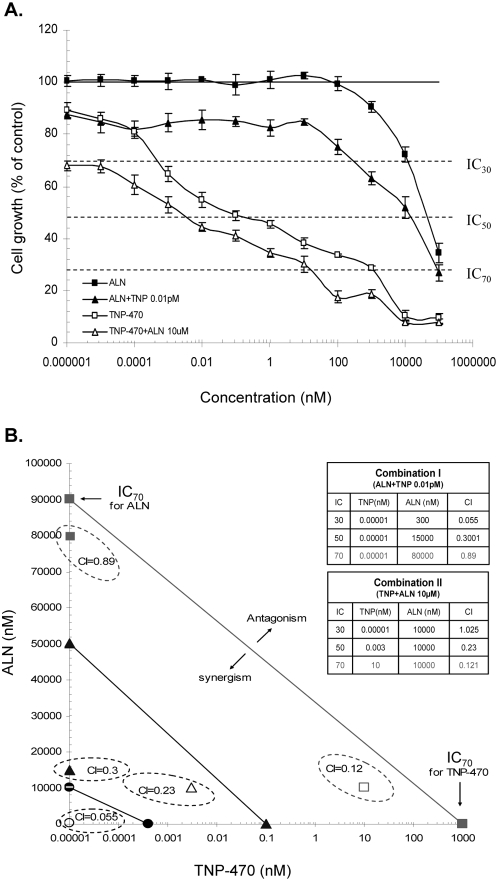
Combination treatment of ALN and TNP-470 has synergistic inhibitory effect on proliferation of endothelial cells *in vitro*. (A) ALN (closed squares) and TNP-470 (open squares) had synergistic effect on HUVEC proliferation when combined as ALN+TNP-470 0.01 pM (closed triangles) and TNP-470+ALN 10 µM (open triangles). (B) Classic isobologram of ALN−TNP-470 combination treatment. IC_70_ (closed squares), IC_50_ (closed triangles) and IC_30_ (closed circles) of ALN and TNP-470. Dashed circles, represent synergism areas of combination. Tables represent the combination index values of each IC of combination treatments I and II. Drug concentration in nM is presented on a logarithmic scale. Data represent mean±s.d.

Prior to conjugation of ALN and TNP-470 to HPMA copolymer backbone, we evaluated the nature of the inhibitory effect of the combination of ALN and TNP-470 on the proliferation of endothelial cells *in vitro*. HUVEC were exposed to free or combined ALN and TNP-470 (Figure 1A). Combination treatments of ALN and TNP-470 decreased the IC of the drugs as single treatments. ALN inhibited cell proliferation at inhibitory concentrations with IC_30, 50, 70_ of 10, 50, 90 µM, and TNP-470 with IC_30, 50, 70_ of 0.00025, 0.1, 1000 nM respectively. Combination treatments I (serial concentrations of ALN and TNP-470 at 0.01 pM) and combination treatments II (serial concentrations of TNP-470 and ALN at 10 µM) inhibited HUVEC proliferation at IC_30, 50, 70_ of 0.2, 10, 30 µM and 0.00001, 0.004, 40 nM respectively. Combination index-isobologram equation allowed quantitative determination of drug interactions, where combination index <1,  = 1, or >1 indicated synergism, additive effect, or antagonism, respectively. Next, data from combination treatments was calculated according to combination index equation and was used to generate isobolograms at IC_30, 50, 70_ of HUVEC proliferation by ALN-TNP-470 combinations (Figure 1B). Combination treatment I had synergistic inhibitory effect on HUVEC at IC_30, 50, 70_ with combination index of 0.055, 0.3, 0.89. Combination treatment II had synergistic effect at IC _50, 70_ with combination index of 0.23, 0.121 and additive effect at IC _30_ with combination index of 1.025.

### Synthesis and characterization of HPMA copolymer-ALN-TNP-470 conjugate

HPMA copolymer-Gly-Gly-Pro-Nle-ALN-TNP-470 conjugate was synthesized in two steps. First, an intermediate was synthesized by copolymerization of HPMA, ALN monomer (MA-Gly-Gly-Pro-Nle-ALN), and amino group containing monomer (MA-Gly-Gly-Pro-Nle-NH(CH_2_)_2_NH_2_). Optionally, for the evaluation of subcellular trafficking, a polymerizable derivative of FITC, *N*-methacryloylaminopropyl (MA-FITC), was added to the monomer mixture. In the second step, TNP-470 was linked to amino groups by nucleophilic substitution of the terminal chlorine of TNP-470 (Scheme S1).

**Figure 2:**
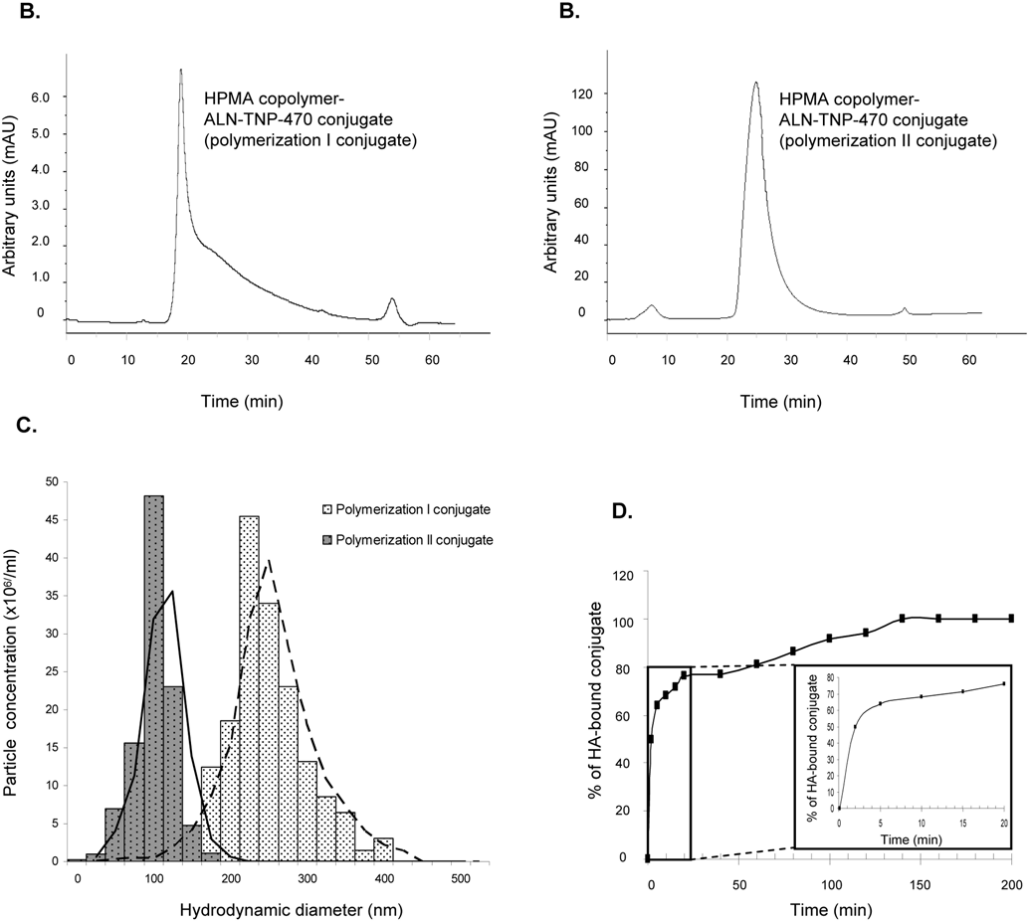
Characterization of HPMA copolymer-Gly-Gly-Pro-Nle-ALN-TNP-470 conjugates. SEC profiles of polymerization I (classical) compound (A) and polymerization II (RAFT) compound (B). Hydrodynamic diameter size distribution (C) of polymerization I compound (gray bars) and polymerization II compound (white bars). (D) Binding kinetics of HPMA copolymer-ALN-TNP-470 conjugate to HA.

The molecular weight and polydispersity of conjugates were estimated by SEC exhibiting an apparent M_w_ of 80 kDa (Figure 2A and 2B). Additionally, the hydrodynamic diameter size distribution of the HPMA copolymer-ALN-TNP-470 conjugates was determined using an optical analyzer. The conjugate polymerized by the classical polymerization method had a PDI of ∼1.62 with a mean size distribution of 241 nm whereas the conjugate polymerized by “living polymerization” RAFT was well-dispersed exhibiting a considerably lower and narrower PDI of ∼1.2 with a mean size distribution of 100 nm (Figure 2C). The values of mean size distribution of the first conjugates, synthesized by the classical polymerization method, clearly indicate that associates formed under the experimental conditions used.

One of the main characteristics of ALN beside its anti-angiogenic and antitumor activities is its pharmacokinetic profile, which exhibits a strong affinity to bone mineral under physiological conditions. To determine if the activity of ALN was retained following polymer-conjugation, we evaluated the affinity of the conjugate to bone mineral by *in vitro* HA binding assay. Following 2 min of incubation, 50% of the conjugate in the solution was bound to HA. This rapid binding rate to HA was decreased after 10 min and finally reached a plateau after 175 min of incubation time with 92% of bound conjugate (Figure 2D).

### Intracellular trafficking of FITC-labeled HPMA copolymer-ALN-TNP-470 conjugate in endothelial and Saos-2 human osteosarcoma cells

**Figure 3:**
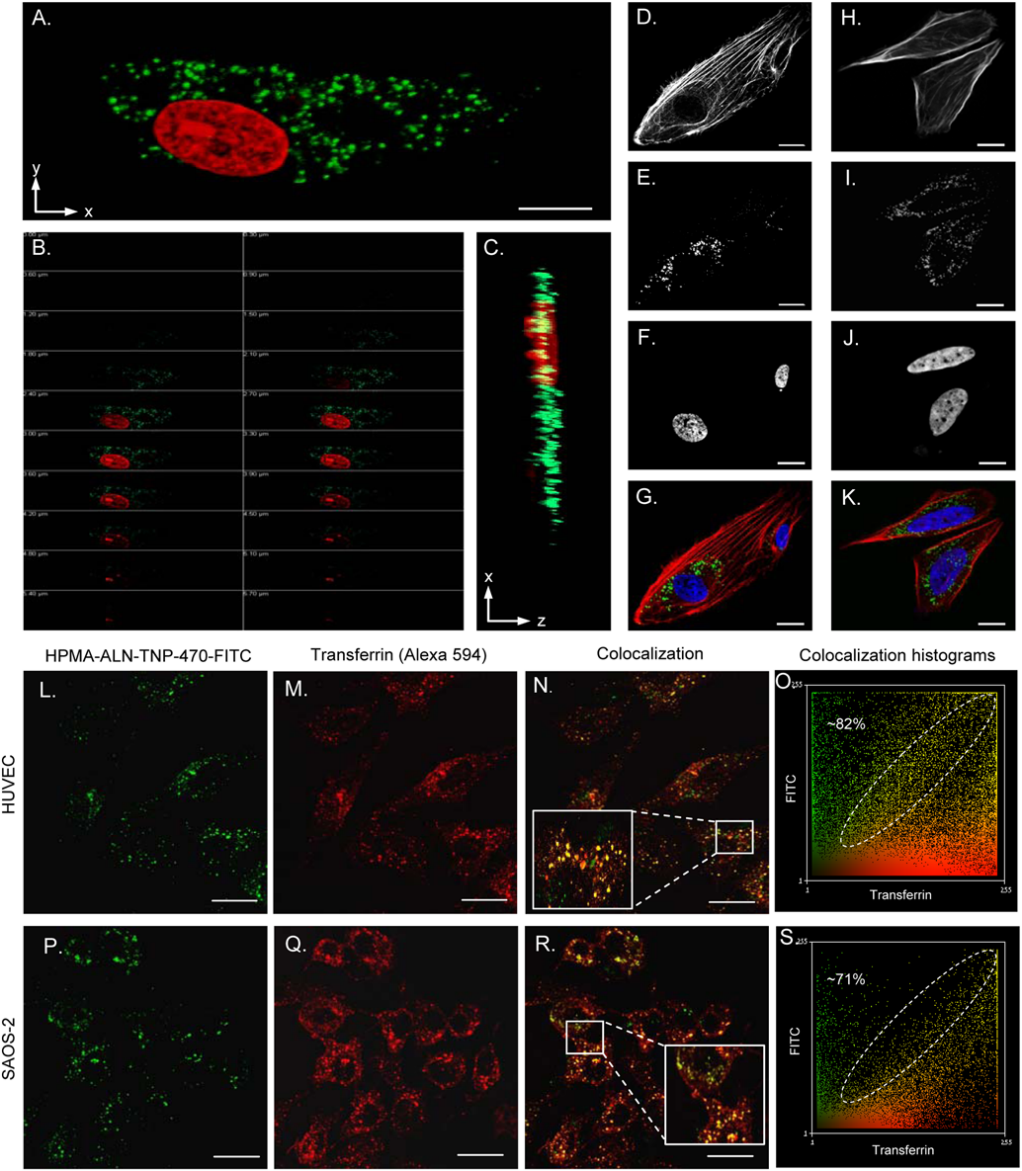
Subcellular trafficking of FITC-labeled HPMA copolymer-ALN-TNP-470 conjugate in HUVEC and Saos-2 human osteosarcoma cells by confocal microscopy. (A) Single XY plane imaging of the conjugate (green) with PI (red) nuclei staining showed cytoplasmatic accumulation of the conjugate. Cellular uptake analysis of the conjugate by HUVEC cells (B) 5.7 µm Z-stack of 28 slices and (C) XZ image slice revealed similar conjugate and nuclei focal plane localization. Multi-channel overlay of (G) HUVEC and (K) Saos-2 cells stained with phalloidin (red) for actin filaments (D, H) and DAPI (blue) for nuclei (F, J) 12 h post incubation with the conjugate (green) (E, I) showed cellular localization of the conjugate mostly around the nuclei. The FITC-labeled conjugate (green) (L, P) colocalized with clathrin-coated vesicles labeled with transferrin (red) (M, Q) in HUVEC (M, N) and Saos-2 (Q, R) cells. Histograms of overlay images revealed a ratio of conjugate/transferrin of 82% in HUVEC (O) and 71% in Saos-2 (S) cells pointing at the cellular uptake of the conjugate via the lysosomotropic pathway. Scale bars represent (A–K) 5 µm and (L–S) 20 µm.

Following chemical characterization, we evaluated the ability of FITC-labeled HPMA copolymer-ALN-TNP-470 conjugate to internalize into endothelial and human osteosarcoma cells and the mechanism by which it enters the cells. HUVEC and Saos-2 osteosarcoma cells were incubated with the conjugate, fixed, permeabilzed and their nuclei were stained with PI. Confocal microscopy performed by multi-channel tracking for PI (red) and FITC-labeled conjugate (green). Following 12 h incubation, conjugate accumulated mostly in the cytoplasm of HUVEC and of Saos-2 cells as observed in the single plane image (Figure 3A). To evaluate the conjugate cellular localization and to eliminate optical artifacts, Z-stack of 5.7 µm with 28 slices (Figure 3B) and X, Z slice (Figure 3C) were captured and analyzed. The conjugate was found to be located at the same focal plane as the nuclei, confirming its intracellular uptake. Further examination of the conjugate cellular internalization was preformed using phalloidin (red) for actin filaments and DAPI for nuclei staining. The staining revealed accumulation of the conjugate mainly around the nuclei in HUVEC (Figure 3D–G) and Saos-2 cells (Figure 3H–K). The conjugate was capable of internalizing into HUVEC and Saos-2 cells as demonstrated by colocalization of 82% of the conjugate with transferrin in HUVEC cells (Figure 3L–O) and of 71% in Saos-2 cells (Figure 3P–S). These high percentages of colocalization suggest a lysosomotropic pathway of cellular uptake via clathrin-coated vesicles.

### HPMA copolymer-ALN-TNP-470 conjugate inhibits the proliferation of endothelial, Saos-2 and MG-63-Ras human osteosarcoma cells

**Figure 4:**
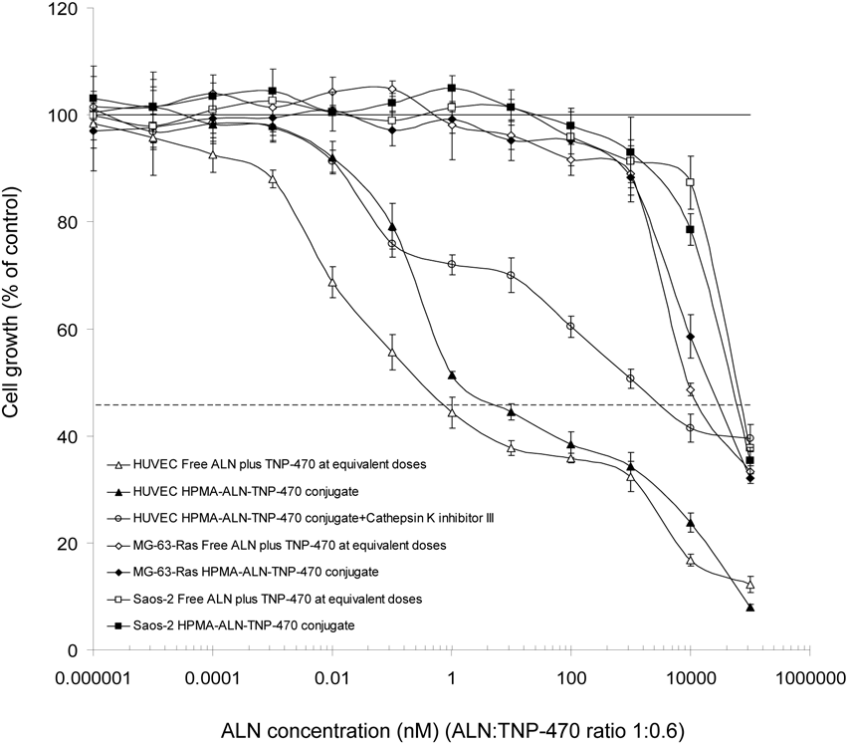
Inhibition of proliferation of endothelial, Saos-2 and MG-63-Ras osteosarcoma cells by HPMA copolymer-ALN-TNP-470 conjugate. Free (open squares/triangles/diamonds) and polymer-conjugated ALN and TNP-470 (closed squares/triangles/diamonds) had similar effect on cell proliferation exhibiting retention of activity following polymer conjugation. Free and conjugated ALN and TNP-470 inhibited ECGS-induced HUVEC proliferation at IC_50_ of 0.7 and 1 nM respectively. Free and conjugated drugs had cytotoxic effect on these cells at doses >1 and 10 nM respectively. The inhibition of endothelial proliferation by the conjugate was reduced significantly (IC_50_ = 4200 nM) in the presence of cathepsin K inhibitor III (open circles). Proliferation of Saos-2 and MG-63-Ras cells was inhibited by free and conjugated ALN and TNP-470 at higher concentrations with IC_50_ of 30 µM and 10 µM respectively. The solid line represents the proliferation of growth factor-induced cells and the dotted line represents the cell proliferation in the absence of ECGS. Data represent mean±s.d. * *P*<0.05, ** *P*<0.03, *** *P*<0.05 compared with control.

Next, we tested the inhibitory effect of HPMA copolymer-ALN-TNP-470 conjugate on HUVEC, Saos-2 and MG-63-Ras human osteosarcoma cell proliferation in order to determine whether it is active *in vitro* and the bound drugs retained their antitumor and anti-angiogenic activity following polymer-conjugation. ECGS-induced proliferation of HUVEC was inhibited similarly by ALN-TNP-470 combination and HPMA copolymer-ALN-TNP-470 conjugate at equivalent concentrations. The free and bound ALN and TNP-470 exhibited an IC_50_ of 0.7 and 1 nM, and had cytotoxic effect at doses higher than 1 and 10 nM respectively (Figure 4). Saos-2 human osteosarcoma cell proliferation was inhibited similarly by free and conjugated ALN-TNP-470 at IC_50_ of 30 µM (Figure 4, A). MG-63-Ras human osteosarcoma cell proliferation was inhibited similarly by free and conjugated ALN-TNP-470 at IC_50_ of 10 µM (Figure 4). HPMA copolymer alone was inert *in vitro* and *in vivo* (data not shown) in agreement with previously published data [57].

In order to validate that HPMA copolymer-ALN-TNP-470 conjugate is active mainly upon the release of ALN and TNP-470 by cathepsin K cleavage mechanism, and not by spontaneous hydrolysis, we evaluated the inhibition of HUVEC proliferation by HPMA copolymer-ALN-TNP-470 conjugate in the presence of cathepsin K inhibitor III (Figure 4). HPMA copolymer-ALN-TNP-470 conjugate inhibited the proliferation of HUVEC at a 4-logs higher concentration in the presence of cathepsin K inhibitor III than in its absence. Following 72 h, there was probably some free ALN and TNP-470 released hydrolytically from HPMA copolymer-ALN-TNP-470 conjugate which led to the inhibition of proliferation of HUVEC at concentrations higher than 4 µM ALN-equivalent concentrations (Figure 4).

### HPMA copolymer-ALN-TNP-470 conjugate inhibits endothelial cells migration towards VEGF

**Figure 5:**
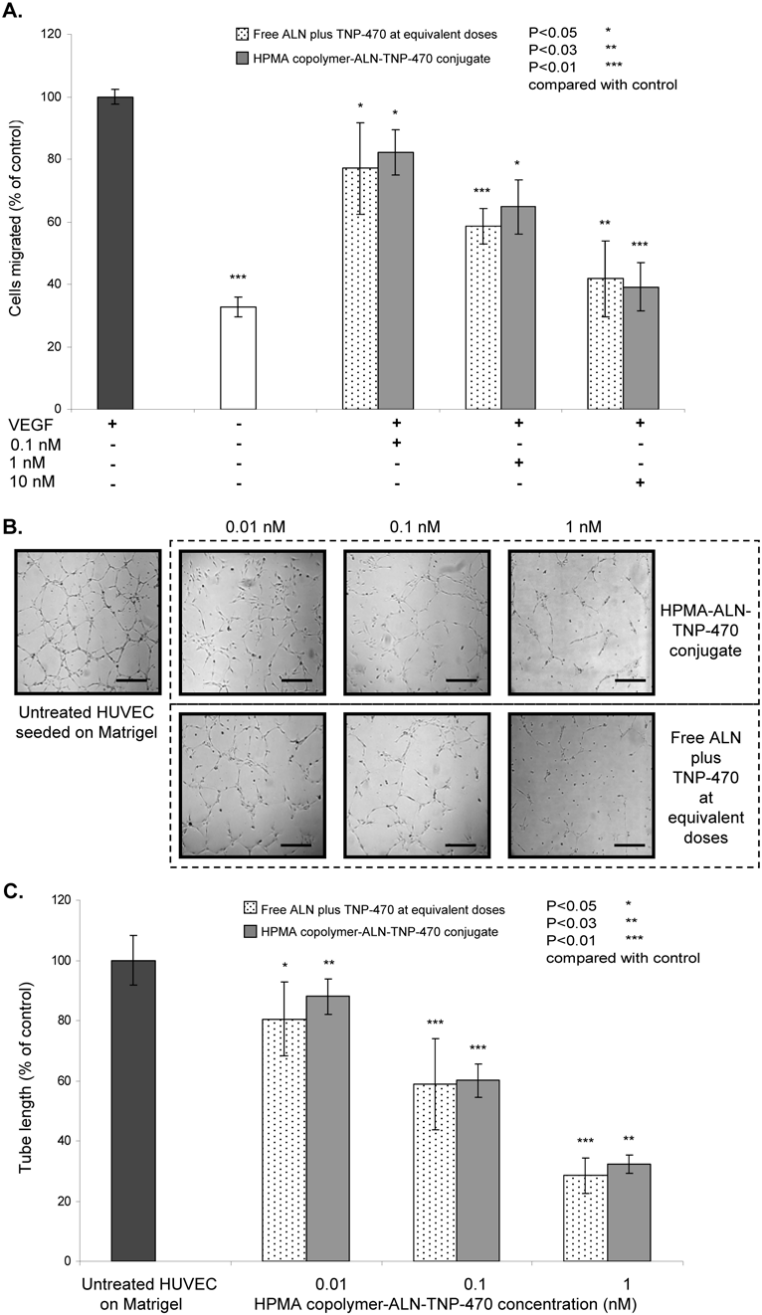
Inhibition of VEGF-induced migration and capillary-like tube formation by HPMA copolymer-ALN-TNP-470 conjugate. Free (dotted bars) and polymer-conjugated ALN and TNP-470 (gray bars) inhibited both (A) VEGF-induced HUVEC migration; and (B, C) capillary-like tube formation of HUVEC, in a dose-dependent manner. Scale bars represent 100 µm. Drug concentration in nM is presented on a logarithmic scale. All *P* values are two-sided (analysis of variance). Data represent mean±s.d. * *P*<0.05, ** *P*<0.03, *** *P*<0.05 compared with control.

We next examined the effect of HPMA copolymer-ALN-TNP-470 conjugate on VEGF-induced HUVEC migration. Migration was assessed by counting the number of cells that migrated through the membranes towards the chemoattractant VEGF during a 4 h period following 4 h treatment with free or conjugated ALN-TNP-470. Treatments with free or conjugated ALN-TNP-470 at equivalent concentrations of 0.1, 1, 10 nM dramatically inhibited the chemotactic migration response to VEGF compared with control (untreated) by 23% (*P* = 0.038), 41% (*P* = 0.003), 58% (*P* = 0.013), and 18% (*P* = 0.037), 35% (*P* = 0.032), 61% (*P* = 0.006) respectively. HUVEC basal migration in the absence of VEGF was 33% compared to VEGF-induced cells (Figure 5A).

### HPMA copolymer-ALN-TNP-470 conjugate inhibits capillary-like tube formation of HUVEC *in vitro*

Having shown that free and conjugated ALN-TNP-470 have anti-angiogenic potential by inhibiting ECGS-induced HUVEC proliferation and migration towards the chemoattractant VEGF, the ability to inhibit capillary-like tube formation of HUVEC was tested. Free and conjugated ALN-TNP-470 at equivalent concentrations of 0.01, 0.1, 1 nM inhibited capillary-like tube length compared with control (untreated) by 34% (*P* = 0.031), 53% (*P* = 0.007), 73% (*P* = 0.004), and 29% (*P* = 0.021), 54% (*P* = 0.008), 64% (*P* = 0.014) respectively (Figure 5B and 5C).

### *In vivo* characterization of HPMA copolymer-ALN-TNP-470 conjugate

Both conjugates synthesized by classical and by RAFT polymerization reactions exhibited similar effects on the *in vitro* assays described above. Therefore, we chose the narrowly dispersed- and smaller in diameter RAFT-polymerized conjugate for all *in vivo* studies due to an expected improved biodistribution.

### HPMA copolymer-ALN-TNP-470 conjugate reduces vascular hyperpermeability

**Figure 6:**
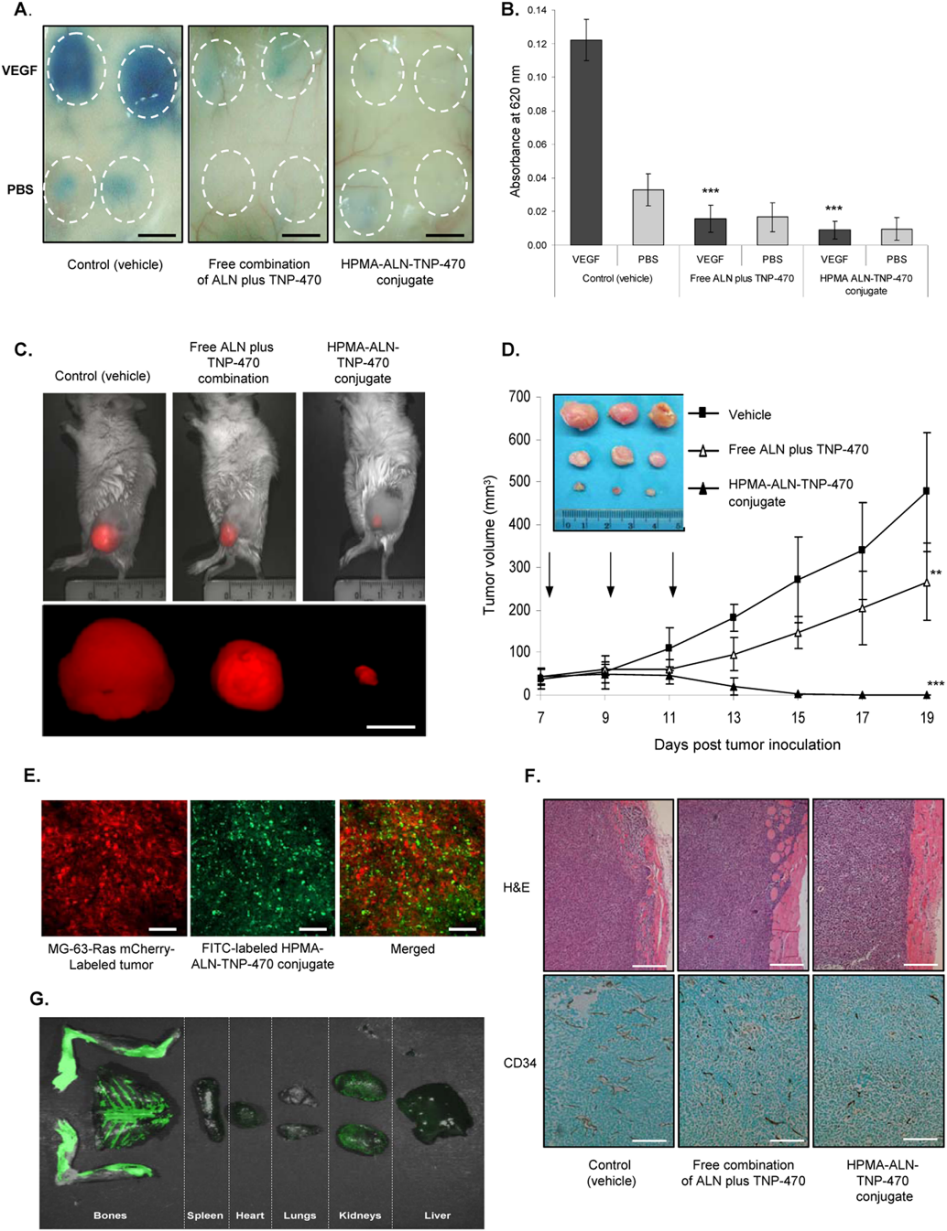
HPMA copolymer-ALN-TNP-470 conjugate reduces vascular hyperpermeability in mouse skin capillaries and inhibits MG-63-Ras human osteosarcoma growth. (A) Diminished dye is observed in mice treated with free or conjugated ALN and TNP-470 compared to untreated control group. Scale bar represents 10 mm. (B) Extracted dye content quantification by measurement at 620 nm. (C) Intravital non-invasive fluorescence imaging of mCherry-labeled MG-63-Ras tumor-bearing mice treated with free or conjugated ALN and TNP-470. (D) Antitumor effect of free (open triangles) or conjugated (closed triangles) ALN and TNP-470 on MG-63-Ras human osteosarcoma tumor growth compared to vehicle-treated group (closed squares) and dissected tumors images. Scale bar represents 10 mm. All *P* values are two-sided. On day 19 HPMA copolymer-ALN-TNP-470 conjugate inhibited tumor growth by 96% (*P* = 0.001) compared with 45% (*P* = 0.012) of free ALN and TNP-470. Data represent mean±s.d. * *P*<0.05, ** *P*<0.03, *** *P*<0.05 compared with control. (E) Whole-mount confocal microscopy of mCherry-labeled MG-63-Ras human osteosarcoma tumors dissected from mice treated with FITC-labeled HPMA copolymer-ALN-TNP-470 conjugate. Scale bar represents 25 µm (n = 5 mice per group). (F) H & E and CD34 staining of control, combination- and conjugate-treated MG-63-Ras osteosarcomas inoculated s.c. in mice. (G) Dissected organs of mice treated with FITC-labeled HPMA copolymer-ALN-TNP-470 conjugate were imaged by the Maestro system showing greater intensity of FITC-fluorescence spectrum (composed images of unmixed multispectral cubes) in bone tissues then in the spleen, heart, lungs, kidneys and liver. Green represent FITC-labeled HPMA copolymer-ALN-TNP-470 conjugate spectrum.

We previously reported that TNP-470 and its conjugated form, caplostatin, reduces VEGF-induced vascular hyperpermeability [14], a prominent early feature of pathological angiogenesis. To determine whether HPMA copolymer-ALN-TNP-470 conjugate is able to reduce microvessel hyperpermeability we used a modified Miles assay. Evans blue dye was injected to the retro-orbital plexus and immediately thereafter the vascular permeability-induced factor VEGF [58] was injected into the shaved flank of Balb/c mice. Evans blue binds to plasma proteins and therefore extravasates along with them at sites of increased permeability [55]. VEGF-induced extravasation of Evans blue dye was remarkably inhibited in mice treated with free combination of ALN and TNP-470 and with HPMA copolymer-ALN-TNP-470 conjugate compared to vehicle treated mice by 87% (*P* = 0.002) and 92% (*P* = 0.001), respectively (Figure 6A and 6B).

### HPMA copolymer-ALN-TNP-470 conjugate inhibits MG-63-Ras human osteosarcoma

SCID male mice bearing s.c. mCherry-labeled MG-63-Ras human osteosarcoma showed decreased tumor growth rates when treated with free and conjugated ALN and TNP-470 (1∶1, 30 mg/kg q.o.d. 3 times). The superiority of administering both drugs when conjugated to the polymer compared to a cocktail of both free drugs becomes evident when injected *in vivo* (Figure 6D). HPMA copolymer-ALN-TNP-470 conjugate exhibited superior antitumor activity compared to free ALN and TNP-470 combination treatment. On day 19 when control mice were euthanized, HPMA copolymer-ALN-TNP-470 conjugate inhibited tumor growth by 96% (*P* = 0.001) compared with 45% (*P* = 0.012) of free ALN and TNP-470 (n = 5 mice per group; Figure 6C and 6D). Confocal microscopy analysis of mCherry-labeled tumors dissected from mice treated with FITC-labeled HPMA copolymer-ALN-TNP-470 conjugate revealed high accumulation of the conjugate at the tumor site (Figure 6E).

H & E staining of tumor sections of MG-63-Ras human osteosarcoma tumors treated with combination of free TNP-470 and ALN or with the conjugate revealed that tumor sections from control mice consisted of poorly differentiated tumor cells invasive through the muscle layer with central calcified areas allowing the HA-targeting of the conjugate with ALN. Conjugate-treated tumors were almost regressed showing cholesterol deposits with connective tissue and giant cells and macrophages around them as signs of regression (Figure 6F). CD34 staining showed reduction of 39% (*P* = 5.5×10^−11^) in microvessel density (MVD) of combination of free ALN with TNP-470 (76±14 microvessels/mm^2^) and 74% (*P* = 4.7×10^−19^) reduction in MVD of conjugate-treated tumors (32±9 microvessels/mm^2^) *vs.* control group of mice (125±16 microvessels/mm^2^) (n = 5 mice per group; Figure 6F).

Fluorescence imaging of organs dissected from mice treated with FITC-labeled HPMA copolymer-ALN-TNP-470 conjugate showed greater intensity of FITC-fluorescence spectrum (composed images of unmixed multispectral cubes) in bone tissues then in the spleen, heart, lungs, kidneys and liver (Figure 6G). Some fluorescence is shown in the kidneys due to renal excretion of the conjugate.

## Discussion

This study describes a new concept of a combination therapy that aims to target angiogenesis-dependent calcified neoplasms. We achieved the synthesis of a conjugate with a predetermined molecular weight with a narrow polydispersity and a high loading of ALN using the RAFT polymerization technique. HPMA copolymer-ALN-TNP-470 conjugate potentially delivers the anti-angiogenic agent by dual targeting of angiogenesis-dependent calcified tumors. ALN actively targets the conjugate to calcified neoplasms whereas size-related passive accumulation is achieved due to the enhanced permeability and retention effect (EPR) typical to tumor blood vessels pathophysiology [19].

We have demonstrated that ALN acts as an anti-angiogenic agent on its own and exhibits enhanced activity while combined with TNP-470 due to their synergistic inhibitory effect on the proliferation of endothelial cells. Furthermore, our nano-conjugate is capable to internalize into the cytoplasm of HUVEC and Saos-2 human osteosarcoma cells via endocytosis. The conjugate inhibited HUVEC proliferation, migration and capillary-like tube formation *in vitro*. In addition, we showed that Saos-2 and MG-63-Ras human osteosarcoma cell proliferation was similarly inhibited by the conjugate and by the combined free drugs demonstrating that the bound drugs retained their antitumor activity following polymer conjugation. As a result, the *in vivo* antitumor efficacy of TNP-470 and ALN have been substantially improved.

A different class of drug-delivery systems bearing a bone targeting moiety has been reported by Hrubý *et al.*, consisting of HPMA copolymer-doxorubicin-hydroxybisphosphonate conjugate bearing a cathepsin B-sensitive linker [59]. The conjugate was tested *in vitro* showing HA binding and drug release. Wang *et al.* described fluorescein-labeled bone-targeted model conjugates for detection purposes bearing 1% loading of ALN or alternatively Asp_8_ on HPMA copolymer [60]. The bone-targeting potential of these conjugates was tested *in vitro* and *in vivo* and was found to selectively accumulate in bone tissue. Here we describe a different polymer conjugation technique, conjugating the ALN to the monomer prior to copolymerization, thus controlling the drug loading and enabling higher loading percentage of ALN (7% mol) on the polymeric backbone. High loading of ALN offers both rapid accumulation in bone tumors and metastases (which produce calcified matter) and enhanced antitumor and anti-angiogenic activity.

Combination therapy is routinely used in conventional chemotherapy [61], [62]. The advantages of polymer therapeutics containing drug combinations have been previously demonstrated [63], [64], [65]. The concomitant delivery of two agents that act synergistically allow the administration of lower concentrations of each drug increasing their combined antitumor efficacy and decreasing their toxicity. However, these examples consisted of the combination of two separate polymer-drug conjugates. Two strategies of combined therapies based on a single polymeric carrier with two drug combinations have been described [59], [66]. The first polymer therapeutic to combine chemotherapy and endocrine therapy was the HPMA copolymer conjugate carrying both the aromatase inhibitor aminoglutethimide (AGM) and doxorubicin for breast cancer therapy [66], [67]. HPMA copolymer–AGM–doxorubicin showed greater cytotoxicity towards breast cancer cells *in vitro* than either of the individual conjugates, or a simple mixture of them. The second combined conjugate synthesized by Hrubý *et al.* was mentioned above. Given that acquired drug resistance is a particular problem for many of the new molecular targets, this new concept provides an interesting opportunity for delivery of two drugs that might act synergistically to block multiple cellular pathways simultaneously. Both combined polymer therapeutics used doxorubicin as the cytotoxic drug and cathepsin B-cleavable linkers. Here, we are targeting the endothelial compartment and the bone tissue bearing the tumor. We have used a cathepsin K-degradable linker, therefore enhancing the specific active targeting to bone cancers.

Combined administration of angiogenesis inhibitors with chemotherapies yield maximal benefits because such combinations destroy two separate compartments of the tumor. Some anti-angiogenic agents can “normalize” the abnormal tumor vasculature, resulting in more efficient delivery of drugs and oxygen to the targeted cancer cells, and enhancement of the efficacy of radiation therapy and chemotherapeutic agents [68]. All of these mechanisms imply that an anti-angiogenic agent would further augment the response to chemotherapy. Despite the relatively good tolerance of angiogenesis inhibitors when administered as single agents, the non-targeted anti-angiogenic drugs often exhibit a non-specific body distribution. This could result in different side effects such as hypertension [69], proteinuria, bleeding, gastrointestinal perforation, arterial thrombotic events [70], exfoliative dermatitis [71] and skin toxicity [72]. Therefore, utilization of combination therapy advantages with targeted drug delivery system is desirable and was taken in consideration when we designed the described conjugate.

The potent angiogenesis inhibitor TNP-470 has been conjugated previously to HPMA copolymer backbone through a Gly-Phe-Leu-Gly-ethylenediamine, a cathepsin B-sensitive linker. Caplostatin exhibited improved accumulation in tumor vasculature due to passive targeting and had reduced toxicity compared to TNP-470. As a result, caplostatin potently inhibited tumor angiogenesis, vascular hyperpermeability and subsequent tumor growth [13], [14], [16], [22]. Our rationale in this study was to synthesize a new polymeric drug delivery system combined of TNP-470 and a strong bone seeking agent such as ALN, to achieve both synergistic inhibitory antitumor effect and dual passive and active targeting effect that could result in a rapid accumulation in bone-associated neoplasms. It was previously shown that subcutaneous administration of TNP-470 and HPMA copolymer-TNP-470 (caplostatin) were effective [13], [14]. Other possible routes of administration of the conjugate are intravenous and intraperitoneal. Most likely, intravenous administration will be the most effective because the conjugate is directly administered into the blood circulation, making the tumor endothelial cells, i.e. the conjugate's target, directly exposed to it.

Polymer-based nanocarriers similar to HPMA copolymer often exhibit inherent structural heterogeneity of the polymers, reflected in a high PDI. Homogenous size distribution of polymer conjugates may contribute to a more defined biodistribution. Here we demonstrate, for the first time, that we have a narrowly dispersed combined antiangiogenic conjugate synthesized by RAFT polymerization. Together with the cathepsin-K-cleavable system we aim to achieve a more specific drug release and therefore focus the toxicity of the free drugs to the bone tumor sites.

It has been shown that ALN inhibits cell survival stimulated by the PI3K/Akt/NFkB pathway via inhibition of the initial step, the activation of PI3K, thus causing apoptosis of osteosarcoma cells [73]. It also inhibits MMP-2 secretion by osteosarcoma cell lines [48]. The synergistic effect of the combination of ALN and TNP-470 might be the consequence of the different mechanisms of action of the drugs.

HPMA copolymer-ALN-TNP-470 substantially inhibited osteosarcoma growth by 96%, whereas combined free drugs demonstrated only 45% inhibition of tumor growth. Immunohistochemical studies showed that HPMA copolymer-ALN-TNP-470 induced reduction of proliferation, invasiveness and angiogenesis. H & E staining indicated a high level of tumor necrosis and signs of regression following treatment. These results suggest that the conjugate is bi-specific by preventing angiogenesis and causing tumor cell death. Mori *et al.* showed that TNP-470 suppressed the pulmonary metastasis of murine osteosarcomas [6]. There is clinical pathological data showing that osteosarcomas preferentially metastasize to the lungs and that these lesions frequently produce calcified bony tissue where osteoid and bone spicules are abundant [74], thus the ALN-bound conjugate could potentially target these metastases as well. Furthermore, the conjugate will target tumors in any site where the vessels are hyperpermeable. Our nanoconjugate will extravasate from those leaky vessels and accumulate in the tumor due to the EPR effect.

The expected advantages of our targeted nano-conjugated polymer therapeutic over the free drugs are (1) longer circulation time due to increased half-life of the drug following polymer conjugation; (2) increased accumulation in tumors compared to normal healthy tissues due to the EPR effect [75]; (3) binding of several BP groups per chain provides a stronger bond to HA delivering higher amounts of TNP-470 or any other drug to the tumor site; (4) elimination of blood brain barrier crossing thus abrogating neurotoxicity; (5) improved antitumor effect due to the fact that TNP-470 and ALN act synergistically as anticancer and anti-angiogenic combination therapy; and (6) solubilization of an insoluble drug. All these advantages led to a significant increase in therapeutic efficacy in an animal model that is highly clinically relevant. This approach warrants the clinical translation of the therapeutic platform described here to be used for calcified neoplasms, bone metastases and other angiogenesis-dependent diseases.

## Supporting Information

Scheme S1 Scheme 1 Synthesis of HPMA copolymer-ALN-TNP470 conjugate.
(1.47 MB TIF)
