# Supplementary figures and images for: Targeting Angiogenesis-Dependent Calcified Neoplasms Using Combined Polymer Therapeutics
Source: PLoS One. 2009 Apr 21;4(4):e5233. doi: 10.1371/journal.pone.0005233 (PMC2667669; doi:10.1371/journal.pone.0005233)

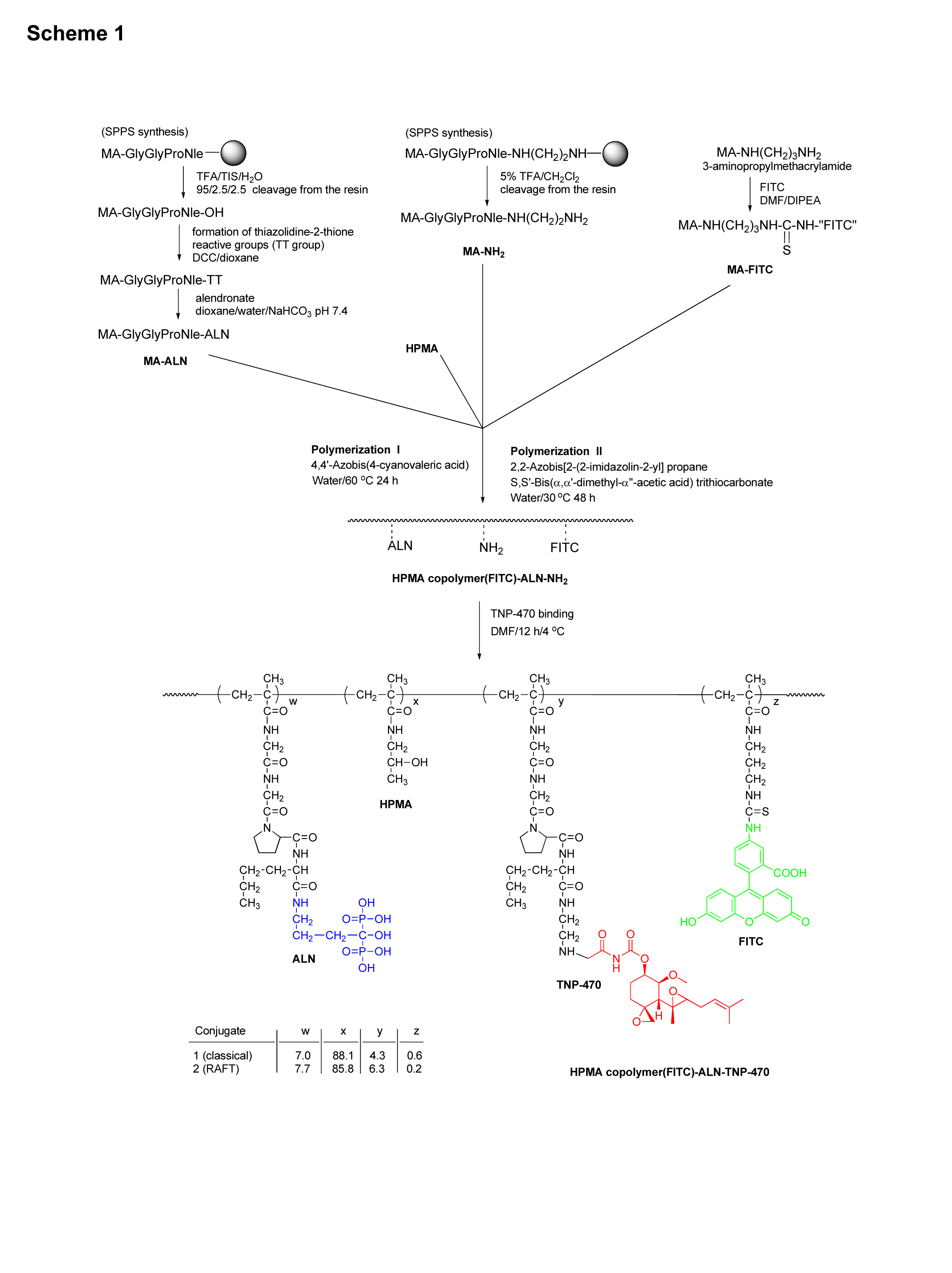

Supplement: Scheme S1 — Scheme 1 Synthesis of HPMA copolymer-ALN-TNP470 conjugate. (1.47 MB TIF) [file pone.0005233.s001.tif]
